# β-Carotene-Induced Alterations in Haemoglobin Affinity to O_2_

**DOI:** 10.3390/antiox10030451

**Published:** 2021-03-13

**Authors:** Joanna Fiedor, Mateusz Przetocki, Aleksander Siniarski, Grzegorz Gajos, Nika Spiridis, Kinga Freindl, Kvetoslava Burda

**Affiliations:** 1 AGH-University of Science and Technology, Faculty of Physics and Applied Computer Science, 30-059 Kraków, Poland; Mateusz.Przetocki@fis.agh.edu.pl; 2 Jagiellonian University Medical College, 31-202 Kraków, Poland; a.siniarski@szpitaljp2.krakow.pl (A.S.); Grzegorz.Gajos@uj.edu.pl (G.G.); 3 The John Paul II Hospital, 31-202 Kraków, Poland; 4 Jerzy Haber Institute of Catalysis and Surface Chemistry Polish Academy of Sciences, 30-239 Kraków, Poland; ncspirid@cyf-kr.edu.pl (N.S.); ncfreind@cyf-kr.edu.pl (K.F.)

**Keywords:** β-carotene, haemoglobin, osmotic fragility, oxygen affinity, red blood cell

## Abstract

β-Carotene (β-Crt) can be dispersed in hydrophobic regions of the membrane of red blood cells (RBC). Its location, orientation and distribution strongly depend on carotenoid concentration. In the present pilot trial (six human subjects involved), it is demonstrated that incubation of RBCs with β-Crt (1.8 × 10^7^ β-Crt molecules per RBC, 50 μmol/L) results in expansion of the membrane of RBCs and slight elongation of the cell. The changes are of statistical significance, as verified by the Wilcoxon test at *p* < 0.05. They indicate (i) a highly random orientation and location of β-Crt inside the membrane and (ii) a tendency for its interaction with membrane skeleton proteins. The accompanying effect of decreased RBC resistance to lysis is possibly a result of the incorrect functioning of ion channels due to their modification/disruption. At higher β-Crt concentrations, its clustering inside membranes may occur, leading to further alterations in the shape and size of RBCs, with the most pronounced changes observed at 1.8 × 10^8^ β-Crt molecules per RBC (500 μmol/L). Due to the reduced permeability of ions, such membranes exhibit increased resistance to haemolysis. Finally, we show that interactions of β-Crt with the membrane of RBCs lead to an alteration in haemoglobin-oxygen affinity, shifting the oxyhaemoglobin dissociation curve toward higher oxygen partial pressures. If the impact of β-Crt on a curve course is confirmed in vivo, one may consider its role in the fine tuning of O_2_ transportation to tissues. Hence, at low concentrations, providing unchanged elastic and functional properties of RBCs, it could serve as a beneficial agent in optimising heart performance and cardiovascular load.

## 1. Introduction

Carotenoids are a ubiquitous group of natural pigments. In biological systems, they fulfil several important functions, among which their antioxidant activity is regarded as the most significant. Carotenoids are built of a series of isoprenoid units that form a polyene backbone that is known to be involved in interactions with reactive oxygen species (ROS) and other free radicals [1]. Hence, they are efficient quenchers of singlet oxygen [2], as well as scavengers of ROS [3], being an important tool against “oxidative stress” and ROS-mediated disorders [4]. From about 700 already known carotenoids, less than 30, including β-carotene (β-Crt), have been found in human blood samples. β-Carotene, being an important dietary compound related to human health, has been subjected to intensive research carried out both in model systems as well as directly in humans. Most of these studies delivered evidence of the advantageous activity of β-Crt. However, it was shown in some model systems under particular experimental conditions [2,5] and in certain population subgroups [6,7] that it may also exhibit an adverse (pro-oxidant) behaviour.

The incorporation of carotenoids into model cell membranes and their impact on the physico-chemical properties of different membrane components have been extensively studied using various biophysical methods [8]. It was shown that carotenoids tend to adopt an extended conformation in lipid bilayers. Some of them were shown to increase the rigidity and stability of membranes and reduce their permeability to ions and oxygen [9,10]. Therefore, we have directed our interests towards exploring the effects of carotenoids on red blood cells (RBCs). The unique membrane skeleton of RBCs ensures the durability and flexibility of these cells, crucial for their proper functioning. Reversible deformation of RBCs is especially important during microcirculation [11]. Any changes in the properties of the membrane skeleton may affect the functioning of RBCs. Under uncontrolled conditions, they may lead to pronounced modifications of cell morphology that further result in the occurrence of undesirable clinical symptoms [12,13]. RBCs consist mainly of haemoglobin (Hb), which is found in the cytoplasm but can largely interact with proteins of the membrane skeleton, especially in its deoxidised form [14,15,16]. Haemoglobin is an α_2_β_2_ heterotetramer with embedded heme moieties. Its main functions (transport of O_2_ and CO_2_, buffering of H^+^ ions, NO metabolism) are already well-established. It is an allosteric protein: binding of one O_2_ to one heme-iron (HFe) increases the O_2_ affinity within the remaining groups. There are many allosteric modulators that affect the Hb-O_2_ binding equilibrium. Apart from well-known pH, *p*CO_2_ and 2,3-diphosphoglycerate, signaling molecules of a different type or natural and synthetic compounds may also induce its shift [17]. Furthermore, the interaction of Hb with the cytoplasmic domain bands 3 of the membrane of RBCs was found to influence the Hb-O_2_ affinity [14].

The above findings prompt us to explore the effect of β-Crt on the stability and functioning of RBCs, with emphasis on the possible changes of the physiological forms of Hb, its structure and molecular properties that may modify Hb-O_2_ affinity. The results were obtained by means of UV-VIS absorption and Mössbauer spectroscopies. The latter is a highly selective technique used for direct determination of the molecular parameters of the HFe and description of the features of its first coordination sphere (conformational and ligand changes) [18,19,20]. The Mössbauer data, along with the osmotic fragility assay, pointed to an unexpected, distinctive effect of β-Crt as a modulator of (i) O_2_-binding properties of Hb and (ii) the membrane features of erythrocytes, such as their stability and permeability to ions, extending our knowledge in this field of science. Finally, the direction of changes is discussed in view of the potential role of β-Crt under hypoxia conditions [21].

## 2. Materials and Methods

### 2.1. Sample Preparation

Fasting blood samples were obtained from six healthy, non-smoking male volunteers 25–35 years old at the John Paul II Hospital in Kraków, Poland. Donors were informed about the scientific purpose of the donation. Their health was verified during a medical examination and by running a series of analytical laboratory tests that included: morphological analysis (white blood cell count, WBC, red blood cell count, RBC, haemoglobin, HGB, haematocrit, HCT, mean corpuscular volume, MCV, mean corpuscular haemoglobin MCH, mean corpuscular haemoglobin concentration, MCHC, red cell distribution width, RDW, platelets, PLT, platelet distribution width, PDW, mean platelet volume, MPV, plateletcrit PCT), quantification of high-sensitivity C-reactive protein (hs-CRP), ions levels (Na^+^, K^+^, Cl^−^), glucose, glycated haemoglobin (HbA1c), creatinine, estimated glomerular filtration rate (eGFR), fibrinogen, lipid profile (TC, LDL, HDL, non-HDL, TG), and liver tests (ALT, AST). After receiving written informed consent to the protocol, 5 mL of donor blood was collected by vein puncture. Sodium heparin was used as an anticoagulant. The blood samples were subjected to further examination within 2 h.

Erythrocytes were isolated by centrifugation (ThermoScientific ST 16R, 3800× *g*, 4 °C, 15 min) and purified by repetitive suspensions in a phosphate buffer (NaH_2_PO_4_/Na_2_HPO_4_, 5 mmol/L, 0.15 mol/L NaCl, pH 7.4) according to the described procedure [22]. The final concentration of RBCs was set to be about 1.65 × 10^9^ cells/mL. The suspension of RBCs was divided into 5 equal fractions containing: (i) control sample (untreated erythrocytes) and (ii–v) erythrocytes treated with different β-Crt concentrations (25 μmol/L, 50 μmol/L, 100 μmol/L and 500 μmol/L, respectively). The applied amounts correspond to ~9 × 10^6^–2 × 10^8^ β-Crt molecules per cell, respectively, whilst the plasma level of β-Crt was reported to be within 10^4^–10^5^ β-Crt molecules per cell (0.21–0.68 μmol/L) [23]. RBCs incubated in the presence of β-Crt are hereafter called as RBCs_x_, where *x* denotes the specific carotenoid concentration.

β-Carotene (95% all-*trans*, Sigma) was solubilised in ethanol and subsequently titrated to the suspensions of RBCs in order to obtain its desired final concentrations. The samples (i–v) were incubated for 10 min at room temperature in the dark. The presence of ethanol did not have any negative effects on the stability and functioning of RBCs. Its final concentration did not exceed 1% *v/v*, which is far from the reported 0.859 mol/L at which echinocyte formation is observed [22,24]. The samples were then washed three times with a phosphate buffer. Finally, each (i–v) was divided into two fractions to test: (a) morphometric parameters and osmotic fragility of RBCs and (b) Hb affinity to O_2_.

### 2.2. Optical Imaging of RBCs

The size and shape of RBCs untreated or incubated in the presence of a given amount of β-Crt were examined using an optical microscope in transmission mode (Olympus IX71, Tokyo, Japan). Images were processed using ImageJ software (National Institutes of Health and the Laboratory for Optical and Computational Instrumentation, University of Wisconsin, Madison, WI, USA). For each sample, 100 randomly distributed cells were chosen, and their average diameters (parameter *d*), as well as longitudinal to lateral ratios (parameter *k*), were determined. To verify statistically significant differences between RBCs subjected to the relevant β-Crt concentrations and their respective controls, a Wilcoxon test at *p* < 0.05 was carried out. Statistical analysis was performed using Statistica 13.1 (StatSoft, Tulsa, OK, USA).

### 2.3. Osmotic Fragility Test

An osmotic fragility test was performed on freshly prepared RBCs (untreated and treated with β-Crt) following a classical procedure [25] with some modifications. Each sample (i–v) was equally divided and added to a series of 30 hypotonic solutions with a NaCl content ranging from 0.9% to 0%, incubated for 10 min at 4 °C, and centrifuged as described above. Absorption spectra of the collected supernatants were recorded in the range of 280–700 nm on a Cary 50 Bio-spectrophotometer (Varian). The haemolysis rate was determined as described previously [26]. The spectra in the range of 460–700 nm were analysed with OriginPro 2019b (OriginLab) using a combination of exponential and Gaussian functions to calculate the area under the spectrum, with the maximum at 577 nm (indicator of the amount of released Hb). The normalised haemolysis curves, as a function of NaCl concentration, are of a sigmoidal character. They were fitted using a basic Boltzmann function.

### 2.4. Mössbauer Spectroscopy

To follow the effect of β-Crt on the reversibility of O_2_ binding to Hb in RBCs, Mössbauer spectroscopy was applied according to the described procedure [13]. The samples were concentrated to a volume of ~1.5 mL (about 8.9 × 10^10^ RBCs per mL). To remove the released Hb, they were washed out three times with a phosphate buffer, as described above. Subsequently, the samples were frozen with liquid nitrogen and stored at −80 °C until use. All laboratory operations were carried out under dim light. Measurements were performed at 85 K in a home-made cryostat enabling gas exchange. The temperature was stabilised within ±0.1 K. 50 mCi ^57^Co(Rh) was used as the source of the 14.4  keV γ-radiation. For each sample, the spectra were collected consecutively every 30 min over 24 h of measurement. The velocity and the isomer shift were calibrated based on the spectrum of the α-iron foil measured at room temperature. Experimental data were analysed using Recoil software [27].

## 3. Results and Discussion

### 3.1. Morphometric and Osmotic Fragility Analyses

**Figure 1:**
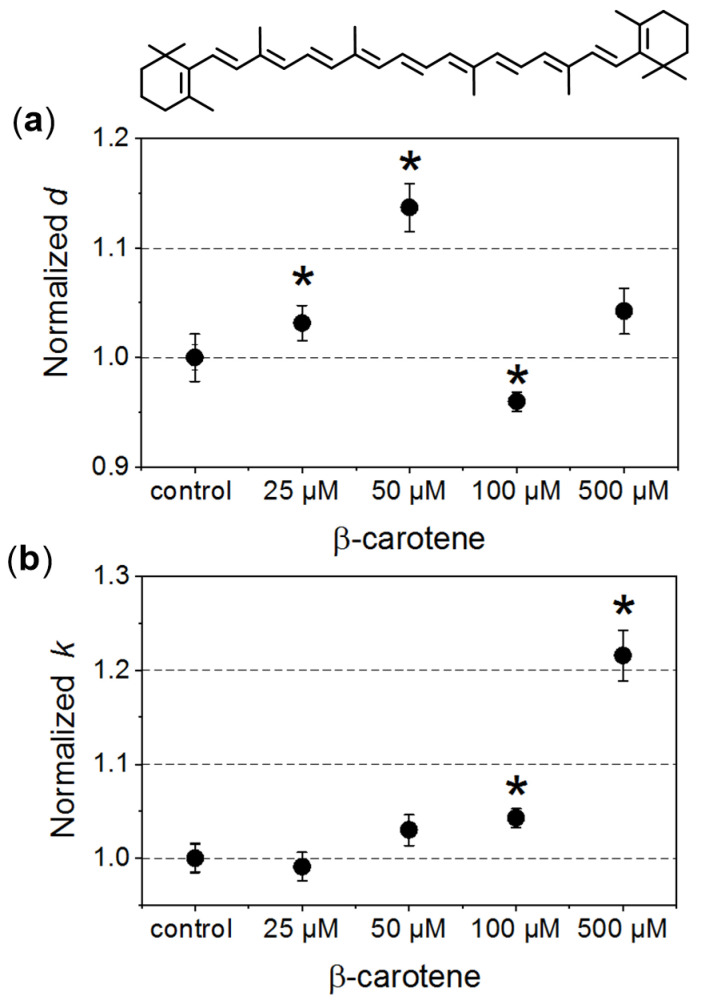
Morphometric changes of red blood cells (RBCs) treated with different β-carotene (β-Crt) concentrations: (**a**) normalised values of diameter, *d*, and (**b**) longitudinal to lateral ratios, *k*. One hundred RBCs per randomly selected donor were analysed. In each case, the mean values of *d* and *k* parameters obtained for three randomly selected donors are shown. The presented errors of the values are calculated from the square of mean standard deviation of the mean average. The statistically significant differences for individual sets of samples (RBC_β-Crt_–RBC_control_), as revealed by the *p*-values (*p* < 0.05) of the Wilcoxon test are marked with an asterisk (*). Top: β-Crt chemical structure.

In our investigated systems, RBCs exhibited a typical biconcave discoid shape. In the case of the control samples, the average diameter of the cells was about 6.5 ± 0.5 μm. In Figure 1, changes of the morphometric parameters obtained for RBCs incubated in the presence of different β-Crt concentrations, designated in relation to their relevant controls, are presented. The average values of normalised diameters (*d*, Figure 1a) and the longitudinal to lateral ratio (*k*, Figure 1b) are shown for each set of samples (i–v) from three independent experiments.

The increase in the mean diameter of RBCs is already observed at a concentration of 25 μmol/L β-Crt, and the parameter *d* reaches its maximum at 50 μmol/L (it is about 15% larger in the case of treated RBCs when compared to control samples). RBCs incubated in the presence of a higher β-Crt concentration (100 μmol/L) are smaller than the control cells; however, an increase in their size is observed again in the presence of 500 μmol/L β-Crt (Figure 1a). It should be noted that the latter effect is associated with a significant elongation of RBCs. In this case, the longitudinal to lateral ratio is by about 20% larger than that estimated for the control samples (Figure 1b). The elongation of RBCs begins already at 50 μmol/L β-Crt.

**Figure 2:**
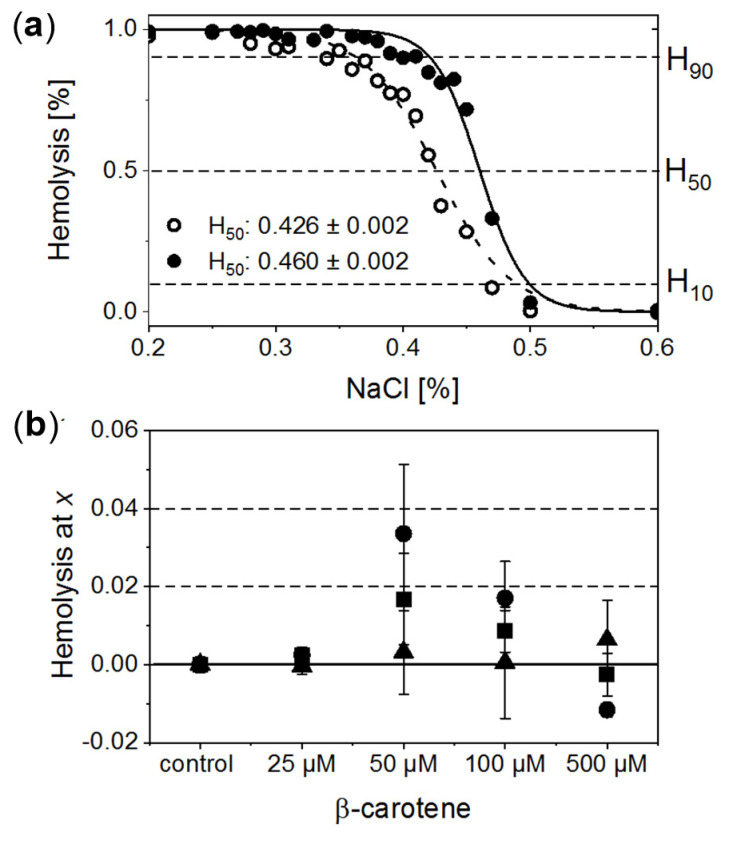
(**a**) Normalised exemplary osmotic fragility curves of RBCs incubated in the presence of 50 μmol/L β-Crt (RBCs_50_, filled circles) accompanied by corresponding control (open circles). Inset: calculated respective H_50_ values. (**b**) Evaluated H_10_ (filled triangle), H_50_ (filled square) and H_90_ (filled circle) as a function of β-Crt concentration. In each case, the mean values from three independently conducted experiments are shown (three randomly selected blood donors). Errors of the values are calculated from the square of mean standard deviation of the mean average. Labels: H_10_, H_50_, H_90_–haemolysis at a level of 10%, 50% and 90%, respectively.

RBCs treated with a series of NaCl solutions (0.9–0%) start to be unstable below a certain salt concentration, and a gradual release of Hb due to the cell membrane disruption is observed. Exemplary osmotic fragility curves are presented in Figure 2a. They have a typical sigmoidal shape. In order to gain quantitative information on haemolysis, the experimental data were fitted with a Boltzmann function. This allowed for estimation of characteristic salt concentrations at which haemolysis: (a) begins (H_10_, haemolysis at a level of 10%); (b) achieves half of the maximum value (H_50_, haemolysis at a level of 50%), and (c) begins to saturate (H_90_, haemolysis at a level of 90%). Comparison of the calculated H_10_, H_50_ and H_90_ values as a function of β-Crt concentration, relative to the corresponding controls, is shown in Figure 2b. Each data point represents an average value of the given parameter obtained from three independent experiments (three randomly selected blood donors).

The analysis of data suggests that β-Crt can modulate the stability and resistance of RBC membranes to osmotic shock. This is manifested by the modification of the shape and position of the sigmoid and, as a consequence, is reflected by the changes of H_10_, H_50_ and H_90_ parameters. H_90_ and H_50_ exhibit a similar dependence on increased carotenoid concentration. Both reach a maximum value at 50 μmol/L β-Crt. H_10_ does not change significantly, although its tendency to increase at the highest used β-Crt concentration, i.e., at 500 μmol/L, is noticeable. Shifts of H_90_ and H_50_ values towards higher concentrations of NaCl point to a decrease in the stability of RBCs (RBCs_50_ and RBCs_100_), while the movement to the opposite direction may suggest an increase in the resistance of RBCs to haemolysis (RBCs_500_). The lowest used β-Crt concentration does not affect the osmotic fragility of RBCs.

To summarise, within the investigated range of β-Crt concentrations the observed size changes of RBCs due to the action of carotenoid correlate with the shift of H_90_ and H_50_ parameters. The maximum of the mean diameter of RBCs treated with β-Crt is found at the carotenoid concentration of 50 μmol/L. At the same β-Crt concentration, a maximum shift of the osmotic fragility curve towards higher concentrations of NaCl is observed. With increasing carotenoid concentrations (>50 μmol/L), the diameter of cells decreases, but at 500 μmol/L, it is again higher compared to the control samples. At a β-Crt concentration ≥ 50 μmol/L, the shape of RBCs undergoes additional modifications, i.e., the cells start to be elongated. The above-described morphological alterations clearly indicate that β-Crt affects the membrane skeleton of RBCs. β-Carotene, as a non-polar compound, can easily incorporate into a cell membrane influencing its thickness, rigidity and inter-leaflet interactions [8,28]. It can freely rotate within the membrane and even flip around the membrane midplane, as was recently shown by molecular dynamics studies [28]. However, the mobility of β-Crt decreases with its increasing concentration. The motility of β-Crt has a noticeable effect on both types of membrane components: lipids and proteins. Therefore, its incorporation into the lipid bilayer of RBCs may influence the organisation and properties of the whole membrane skeleton. Our data is in line with recent results showing that at low β-Crt concentrations (up to 5 mol%), its motility can lead to local distortions in the lipid orientation and results in a less rigid membrane structure, making it more susceptible to deformations [28]. The presence of β-Crt, apart from the perturbation of the physical and chemical properties of the protein-lipid bilayers, results in an alteration in the functioning of ion channels, as observed by the reduced resistance of RBCs against haemolysis. However, a further increase of β-Crt concentration could result in its aggregation, as its elevated concentrations are known to make the membrane thicker and more rigid [28,29] and, as a consequence, less permeable to ions. This can be apparently seen at 500 μmol/L β-Crt, at which the osmotic fragility curves shift back toward lower NaCl concentrations. At β-Crt concentrations >50 μmol/L, its enhanced interactions with the internal membrane structure are clearly seen (Figure 2). As previously mentioned, they are responsible for a serious rearrangement/disruption of the membrane skeleton network. Similar changes of the shape of RBCs were observed for hypertensive erythrocytes [13], as well as other pathological conditions [30,31].

### 3.2. Mössbauer Spectroscopy

Mössbauer spectroscopy is highly sensitive to the valence and spin states of the probe atom (in this case, the ^57^Fe-isotope naturally occurring in heme) and its chemical surrounding in its first coordination sphere. The hyperfine parameters, an isomer shift (IS) and a quadrupole splitting (QS), characterise the HFe and thus the state of Hb [18,32]. Mössbauer experiments were carried out only for those β-Crt concentrations which caused significant changes in the morphology and stability of RBCs. The contributions of the respective Hb phases in the samples were calculated based on the relative area of the corresponding doublet in the entire absorption spectrum area.

**Figure 3:**
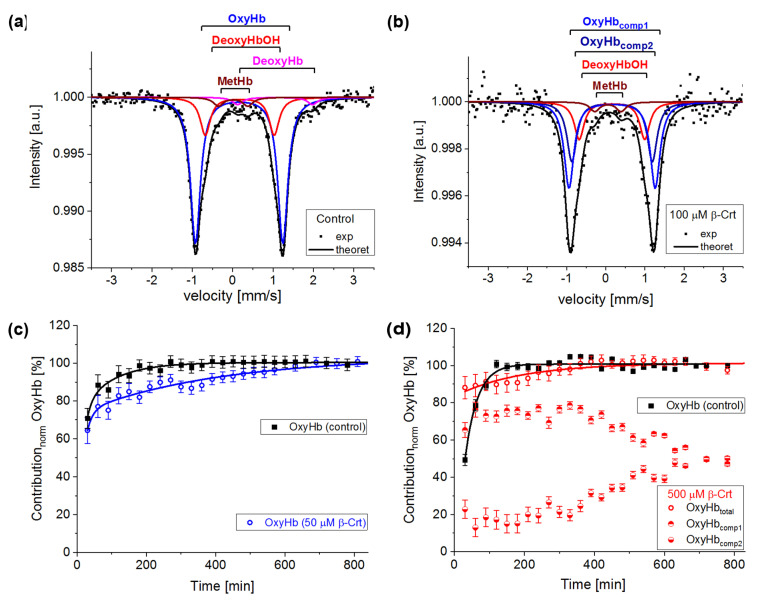
Exemplary steady-state Mössbauer spectra of RBCs: (**a**) untreated (control), and (**b**) incubated with 100 μmol/L β-Crt (RBCs_100_) measured at 85 K. The experimental data (exp) is indicated by points and theoretical curves (-theoret) by lines. The characteristic doublets for the different forms of haemoglobin are shown as sub-spectra. Time evolution of the normalised oxyhaemoglobin contribution in RBCs incubated in the presence of 50 μmol/L β-Crt (blue open circle) (**c**) and 500 μmol/L β-Crt (red open circle) (**d**), in comparison with respective controls (filled square). Additionally, in (**d**), two resolved subcomponents of oxyhaemoglobin in RBCs_500_ are shown (OxyHb_comp1_, half-up filled circle; OxyHb_comp2_, half-down filled circle). The oxygen saturation level of Hb in the treated and corresponding untreated RBCs was the same within the measurement uncertainty: for RBCs_50_ ~73 ± 1% and RBCs_500_ 72 ± 2%. Error bars shown in Figures (**c**,**d**) correspond to the accuracy of the theoretical fit of Mössbauer spectra.

In Figure 3a, an exemplary spectrum of a control sample treated as described in [13] is presented. In order to get a good fit of this spectrum, four components had to be taken into account: oxyhaemoglobin (OxyHb, haemoglobin saturated with O_2_, low spin HFe^2+^), deoxyhaemoglobin (DeoxyHb, physiological Hb with six coordinated high spin HFe^2+^ axially bound to histidines), non-physiological deoxyhaemoglobin (DeoxyHbOH, the haemoglobin with hemes having OH/H_2_O as the sixth ligand, HFe can be in a mixed spin and valence state Fe^2+^/Fe^3+^) and methaemoglobin (MetHb, the haemoglobin with five-coordinated HFe^3+^), [18,32]. In the investigated samples, OxyHb is characterised by IS = 0.15 ± 0.03 mm/s and QS = 2.13 ± 0.04 mm/s, DeoxyHb by IS = 0.95 ± 0.15 mm/s and QS = 2.15 ± 0.15 mm/s, DeoxyHbOH by IS = 0.16 ± 0.03 mm/s and QS = 1.6 ± 0.1 mm/s and MetHb by IS = 0.08 ± 0.06 mm/s and QS = 0.55 ± 0.15 mm/s. The same forms of Hb are observed in samples incubated with β-Crt. However, DeoxyHb is not always visible in Mössbauer spectra, as it is known to usually convert to OxyHb within the first two hours of measurement, or its content is not high enough to be detected. Interestingly, in the spectrum of RBCs_100_ and RBCs_500,_ a new component appears. In RBCs_100_, it becomes detectable after four hours of measurements, whereas in RBCs_500_, it can be resolved from the start (Figure 3d). The IS and QS of this component are smaller by about 0.02 mm/s and 0.12 mm/s, respectively than the corresponding hyperfine parameters of OxyHb in control samples. The hyperfine parameters of this component and its increasing contribution to the spectrum at the expense of OxyHb, along with lengthening of the measurement time, suggest that it is oxyhaemoglobin, in which the symmetry of the binding of HFe to O_2_ is changed. This can be related to both the length of the Fe-0 binding and its motility. We called it OxyHb_comp2_, while the disappearing component was named OxyHb_comp1_ (Figure 3b,d).

**Figure 4:**
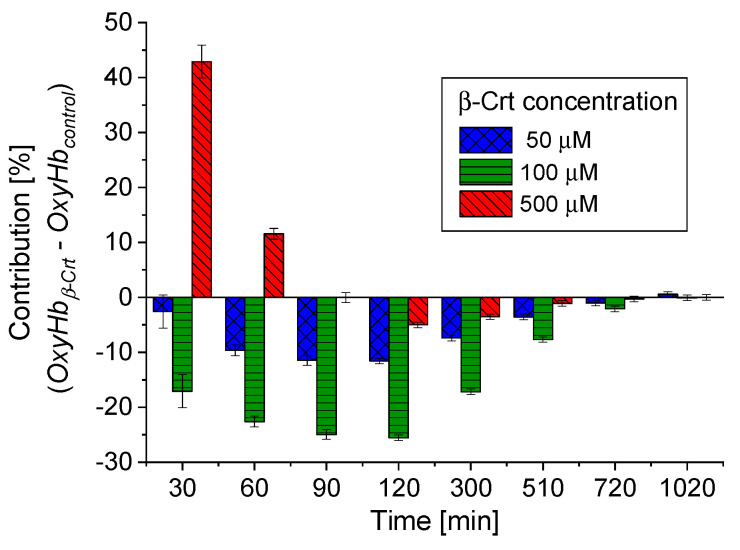
Comparison of time-dependent changes in OxyHb contributions in RBCs treated with different β-Crt concentrations, with respect to corresponding controls. The data is derived from Mössbauer spectra collected during certain time periods. Characteristic periods were selected to best visualise the differences in the variability of OxyHb_β-Crt_—OxyHb_control_ for the β-Crt concentrations indicated. Average square errors are shown.

According to the applied procedure, the time-dependent Mössbauer measurements allowed us to monitor the reversibility of oxygen molecule binding by deoxygenated Hb inside the RBCs. Under the experimental conditions, each sample was incubated at high N_2_ pressure at room temperature for 30 min, which facilitated the release of O_2_ from Hb in RBCs. Transfer of the sample to low N_2_ pressure conditions at 85 K enabled O_2_ rebinding by Hb. The low temperature slowed down all diffusive and enzymatic processes. The time evolution of OxyHb in RBCs incubated at 50 μmol/L and 500 μmol/L β-Crt is shown in Figure 3c,d, respectively. These figures also contain data for OxyHb in appropriate control samples to demonstrate the effect of β-Crt on Hb saturation with O_2_. Additionally, for RBCs_500_, besides the time evolution of total OxyHb (OxyHb_total_), its two subcomponents, OxyHb_comp1_ and OxyHb_comp2_, are shown. One sees that the process of O_2_ saturation by Hb is much slower in RBCs treated with β-Crt. In RBCs_50_ and RBCs_500_, OxyHb reaches a steady state level after about 800 min and 400 min, respectively (Figure 3c,d). For better visualisation of the differences between OxyHb saturation kinetics in RBCs treated with β-Crt (50 μmol/L, 100 μmol/L and 500 μmol/L) and in their corresponding control samples, the differences between the contributions of OxyHb in Mössbauer spectra collected during the particular time periods are shown in Figure 4. In RBCs_100_, OxyHb reaches its stable level even slower than in RBCs_50_. The kinetic of OxyHb saturation is noticeably faster in RBCs treated with 500 μmol/L than in those incubated with lower applied β-Crt concentrations. Furthermore, in RBCs_500_, the change in the OxyHb content measured during time periods <90 min with respect to that observed in its corresponding control sample is positive (OxyHb_β-Crt_—OxyHb_control_ > 0), contrary to RBCs_50_ and RBCs_100_ (Figure 4). This suggests that Hb in RBCs_500_ may re-bind the oxygen molecule at much lower O_2_ partial pressure than in the case of the control sample. In RBCs treated with lower concentrations of β-Crt, OxyHb is formed at higher O_2_ partial pressure than in the control samples.

**Figure 5:**
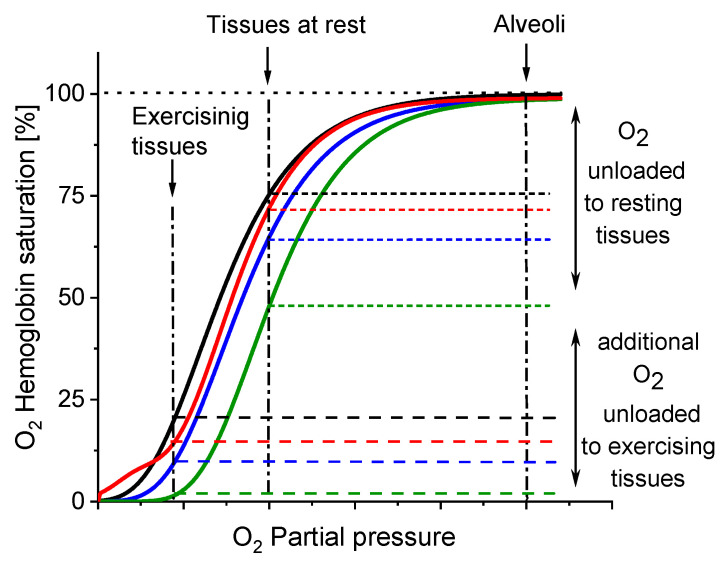
Direction of changes in Hb-O_2_ affinity as a result of β-Crt impact on RBCs. The oxyhaemoglobin dissociation curves (ODCs) are based on the theoretical evaluation of OxyHb saturation changes observed in the time dependent Mössbauer experiments (see: Figure 3c,d and Figure 4). Labels: control (black), RBCs_50_ (blue), RBCs_100_ (green), RBCs_500_ (red).

These effects, reflected in the oxyhaemoglobin dissociation curve (ODC), show the direction of changes in Hb affinity to O_2_ due to increased β-Crt concentrations in RBCs (Figure 5). It should be noted that the oxygen saturation level of Hb in the treated and corresponding untreated RBCs was the same or very similar (see legend in Figure 3c,d), meaning that there has been no reduction in the amount of OxyHb in RBCs incubated with β-Crt. Under applied experimental conditions, temperature and Bohr effects can be excluded. Therefore, the right shift of the ODC associated with reduced Hb-O_2_ affinity may suggest modification of Hb as a result of its increased affinity to 2,3-bisphosphoglycerate or other organic phosphates [33]. On the other hand, one cannot exclude the direct influence of β-Crt on the binding of O_2_ by Hb. Obviously, there are a variety of factors that can be responsible for the ODC shifts, for example, ATP or Mg^2+^ and Cl^−^ ions [34], and β-Crt can be considered as one of them.

The results demonstrate that β-Crt may alter a highly specific interaction of Hb with the membrane skeleton protein(s). So far, it was shown that the interaction between DeoxyHb and band 3 protein is O_2_-dependent, and the cytoplasmic domain of band 3 (cdb3) was proved to be responsible for the shift of the ODC toward higher O_2_ partial pressures [15,16]. Here, under physiological conditions, decreasing the Hb-O_2_ affinity in β-Crt treated RBCs should increase the supply of O_2_ to resting and exercising tissues (Figure 5). The greatest and least effects were observed for RBCs_100_ and RBCs_500_, respectively. In the latter case, a new phenomenon is present, namely an increased Hb-O_2_ affinity at very low oxygen partial pressures in comparison to the control sample (Figure 3d, Figure 4 and Figure 5). It is worth noting that this phenomenon is accompanied by the most pronounced change in the shape of RBCs_500_ and decreased permeability of their membrane for Na^+^ ions (Figure 1 and Figure 2b). Elongation of RBCs was also observed in cells with impaired interactions between ankyrin and band 3 as a result of mutations in the cdb3 domain [35,36]. Similar morphological changes were also found in RBCs from patients with untreated essential hypertension. However, in this case, the ODC shifted to lower O_2_ partial pressures in comparison to healthy RBCs [13]. It was suggested that an alteration of interactions within the ankyrin-spectrin and/or the adducing-actin–spectrin complexes could result in an opposite effect, i.e., a left shift of the ODC. Hence, the more complex course of the ODC in RBCs_500_ can be explained in two ways: (i) at not enough high oxygen partial pressure, Hb molecules exhibit negative cooperative binding of O_2_, and this may turn into positive cooperation when sufficiently high oxygen pressure is reached but remains lower than in the control sample, and (ii) in some Hbs, one bound O_2_ molecule reduces their affinity to other oxygen molecules, while in others, the cooperative binding of O_2_ is positive. In both cases, the Hill factor is lower than in the control sample. Regardless of the scenario, it can be expected that disorder in the organisation and functioning of the membrane skeleton of RBCs caused by β-Crt is, among other things, responsible for the observed effects.

In fact, reducing the affinity of Hb to O_2_ favours the release of oxygen from Hb into the tissues and thus results in a decrease in the circulatory load. It has been reported that such a phenomenon is beneficial, for example, in hypoxia adaptation at high altitudes but only up to about 5500 m. At higher altitudes, increased Hb-O_2_ affinity is desired to preserve arterial O_2_ saturation and ensure oxygen supply to tissues under conditions of critically low oxygen partial pressure in cells [37,38]. Interestingly, the same effects have been recently reported in the case of patients with severe pulmonary disorders such as coronavirus disease 2019 (COVID-19) [21]. A significantly higher Hb-O_2_ affinity has been observed during severe prolonged hypoxia. However, in about 10% of COVID-19 cases, lower Hb-O_2_ affinity has been detected, which may suggest less severe hypoxia and/or a shorter period of its duration in these patients.

In view of the above findings, β-Crt may turn out to act as a positive agent in the modulation of interactions between Hb and O_2_, facilitating adaptation to hypoxemia. On the other hand, its pro-oxidant potential should be kept in mind. The observed changes in Hb-O_2_ affinity upon β-Crt action may lead to local changes in the oxygen level. Enhanced delivery of oxygen to resting and/or exercising tissues may result in oxidative stress and formation of reactive derivatives. Although β-Crt is known to act as an efficient antioxidant in biological systems, there are some reports on its disadvantages, e.g., a carcinogenic response as a result of the elevated β-Crt presence, that were associated with the formation of β-Crt metabolites (its short-chain and long-chain cleavage products) [39,40]. In our case, the structural modification of β-Crt upon increased oxygen levels cannot be excluded. Hence, a formation of its oxidative breakdown products of pro-oxidant activity may take place that could further affect the morphology, stability and functionality of RBCs.

The concentration of β-Crt used in our experiments (25–500 μmol/L) is about two orders of magnitude higher than the level usually detected in plasma (0.21–0.68 μmol/L) [23], which correspond to the average intake of β-Crt of 1–5 mg/day (or up to 10 mg/day depending on seasonal and regional variations) [41]. β-Carotene is delivered in form of food or supplements; however, no upper level of its intake has been established [41]. There are many reports of the administration of high doses of β-Crt (≥30 mg/day) for a long time which did not cause side effects in healthy people, although this resulted in its increased plasma concentration. The mean plasma concentration of β-Crt was more than 3 times higher compared to the control group, even after a short (9 days) β-Crt enriched diet (~40 mg/day) [42]. Moreover, in vivo studies showed that oral administration of an increased amount of β-Crt (580 mg/day for one week) resulted in an about 36-fold (~13 μmol/L) and a 7-fold increase in its concentration in plasma and RBCs, respectively [43]. Hence, the lower concentrations of β-Crt employed in our experiments can be reached in vivo by its high dietary intake such as via supplements.

In the present study, β-Crt was shown to alter the resistance of RBCs membranes to osmotic shock and affinity of Hb to O_2_. The experiments were performed on material taken from healthy donors. However, due to the limited number of analysed cases, these results should be treated as a promising pilot trial. Before this observation can be considered fully confirmed, further systematic studies revealing the mode of β-Crt action on RBCs from healthy individuals and donors with respiratory system diseases are needed.
